# A mathematical model to predict the requirement for multiple Z plasties

**DOI:** 10.4103/0970-0358.41129

**Published:** 2008

**Authors:** Sunderraj Ellur

**Affiliations:** Department of Plastic Surgery, St Johns Medical College Hospital, Bangalore, Karnataka, India

Dear Sir,

It is Well Known That In a 60° Z plasty, the contractural diagonal is placed perpendicular to the other diagonal of the parallelogram and the length of the contractural diagonal equals the limbs of the Z[1] plasty.

The contractural diagonal lengthens by 75% when the two Z plasty flaps are transposed.[2] The flaps transpose ONLY if there is adequate lax tissue in the areas adjacent to the contractural diagonal. Hence, in effect, the lengthening across the contractural diagonal is accompanied by shortening of the diagonal at right angles to it. In a clinical situation, this shortening may be limited by tissue rigidity causing limitation in the size of the Z plasty. One can assess the laxity of the tissues by pinching skin between the thumb and the fingers.

Below we describe a mathematical theorem to deduce the size of a Z plasty from the measurements of the laxity of the tissues adjacent to the Z plasty.

**Figure 1:**
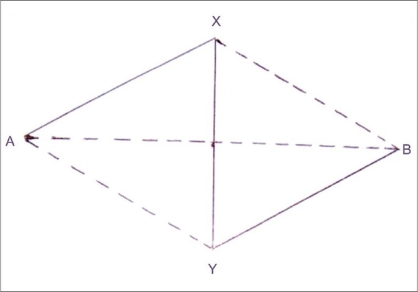
XY is the scar and the contractural diagonal of the planned Z plasty, AX-XY-YB

In Figure 1, XY represents the scar.

Let the contractural diagonal marked over the scar XY, which is equal to the limbs, AX and YB of the designed Z plasty, be equal to C cm. AB represents the other diagonal of the parallelogram, which also represents the points adjacent to the scar where shortening happens after transposition of the flaps. Let AB be equal to T cm.

**Figure 2:**
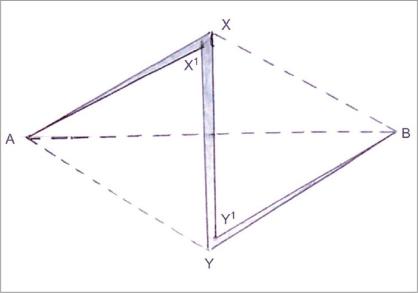
Incision along the planned Z resulted in two triangular flaps AX′Y and By′X

In Figure 2, incisions made along AX, XY and YB will result in two triangular flaps AX′Y and BY′X, which are ready to be transposed. Note: AX = AX′ = X′Y = XY′ = YB = Y′B = C cm.

**Figure 3:**
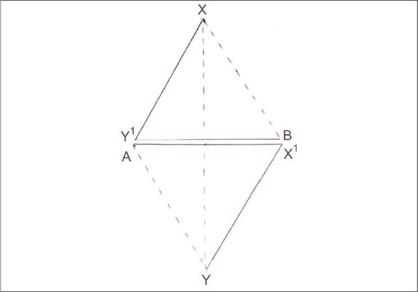
Flaps transposed and inset

Figure 3 shows the transposed flaps in which inset sides Y′B, AX′ each equal to C cm lie side by side. Notice that the distance between the points AB, which was T cm before transposition of flaps is now C cm. Hence, the shortening, which may be designated as L for laxity is T – C cm.

Hence, L = T – C

In Figure 1, if the intersection of the two diagonals is represented as I, then the triangle AIX is a right-angled triangle.

According to Pythagoras' theorem, (AX)^2^ = (AI)^2^ + (IX)^2^.

AX = C. Since I is the center of AB, AI = T/2. Since I is the centre of XY, IX = C/2.

$$\begin{array}{ccc} \text{Hence},{\text{C}}^{2} & \text{=} & {\text{(T/2)}}^{2}+{\text{(C/2)}}^{2}\\ {\text{(T/2)}}^{2} & \text{=} & {\text{C}}^{2}-{\text{(C/2)}}^{2}\\ {\text{(T/2)}}^{2} & \text{=} & {\text{C}}^{2}-{\text{C}}^{2}\text{/4}\\ {\text{(T/2)}}^{2} & \text{=} & (¾){\text{ C}}^{2}\\ \text{T/2} & \text{=} & \text{C√3/2}\\ \text{T} & \text{=} & \text{C√}3 \end{array}$$

We have deduced above that laxity, L = T – C

$$\begin{array}{ccc} \text{Hence},\text{L} & \text{=} & \text{C}\surd 3-\text{C}(\surd 3\text{=}1.73)\\ \text{L} & \text{=} & 1.73\text{C}-\text{C}\\ \text{L} & \text{=} & 0.73\text{C}\\ \text{Hence},\text{C} & \text{=} & \text{L}/0.73 \end{array}$$

To summarize, if the laxity can be assessed and estimated by the pinch test as L then L divided by 0.73 gives the size of the Z plasty that must be used.

Examples:

If Laxity, L = 2 cm, the size of Z plasty, C must be 2 divided by 0.73 = 2.74 cm or less.

If L = 1 cm, the size of Z plasty, C must be 1 divided by 0.73 = 1.34 cm or less.

If L = 3 cm, the size of Z plasty, C must be 3 divided by 0.73 = 4.11 cm or less.

To conclude, irrespective of the length of the scar, the size of the Z plasty drawn should be based on the laxity available. If there is insufficient laxity, the contracture will require more than one Z plasty to lengthen the full length of the contracture. If the tissue laxity [L] is ≥ the length of the scar, a single Z plasty is required. By this method the surgeon can decide whether a single or multiple Z plasties are required.

However, the clinical decision whether to use single or multiple Z plasties is determined by two other factors - whether a single or multiple Z plasty is more aesthetic and whether there is a confounding immovable anatomical feature (e.g., scar revision around the nose). Further since the skin doesn't behave as a mathematical model, one needs to find a method to accurately measure the amount of laxity in the skin.
